# In Vitro Evaluation of Ethanolic Extracts of *Ageratum conyzoides* and *Artemisia absinthium* against Cattle Tick, *Rhipicephalus microplus*

**DOI:** 10.1155/2014/858973

**Published:** 2014-12-01

**Authors:** S. Parveen, R. Godara, R. Katoch, A. Yadav, P. K. Verma, M. Katoch, N. K. Singh

**Affiliations:** ^1^Division of Veterinary Parasitology, Faculty of Veterinary Sciences and Animal Husbandry, Sher-e-Kashmir University of Agricultural Sciences and Technology, R.S. Pura, Jammu 181 102, India; ^2^Division of Veterinary Pharmacology and Toxicology, Faculty of Veterinary Sciences and Animal Husbandry, Sher-e-Kashmir University of Agricultural Sciences and Technology, R.S. Pura, Jammu 181 102, India; ^3^Indian Institute of Integrative Medicine (CSIR), Canal Road, Jammu 180 001, India; ^4^Department of Veterinary Parasitology, College of Veterinary Science, Guru Angad Dev Veterinary and Animal Sciences University, Ludhiana 141 004, India

## Abstract

In vitro efficacy of ethanolic extracts obtained from the aerial parts of *Ageratum conyzoides* and *Artemisia absinthium* was assessed on *Rhipicephalus microplus* using adult immersion test (AIT). Five concentrations of the extract (1.25%, 2.5%, 5%, 10%, and 20%) with three replications for each concentration were used in the bioassay. In AIT, the maximum mortality was recorded as 40% and 66.7% at 20% concentration for *A. conyzoides* and *A. absinthium*, respectively. Acaricidal activity was found to be higher in the extract of *A. absinthium* with LC_50_ and LC_95_ values of 11.2% and 61.7%, respectively. Egg mass weight of the live ticks treated with different concentrations of the extracts was significantly (*P* < 0.05) lower than that of control ticks; consequently, the reproductive index and oviposition values of the treated ticks were reduced significantly (*P* < 0.05). The *A. conyzoides* inhibited 90% hatching of eggs at the 20% concentration, whereas *A. absinthium* showed 100% inhibition at 5%, 10%, and 20% concentrations. The results show that *A. absinthium* has better acaricidal properties than *A. conyzoides* and could be useful in controlling *R. microplus*.

## 1. Introduction

The parasitic diseases are a global problem and are considered a major obstacle in the health and product performance of animals. Among ectoparasites, the ticks are very important and are harmful blood sucking parasites of livestock. The impact of ticks on human economy merits special consideration as they affect the health of domestic wealth directly and indirectly. Although they are mainly recognized as pests, ticks are best known for their notorious vector status [1]. The tick bite marks diminish up to 20–30% of the value of skins and hides [2]. In India alone, the cost of tick and tick-borne diseases (TTBDs) and tick worry in animals has been estimated in the tune of 498.7 million US $ and 57.2 million US $ per annum, respectively [3]. According to FAO [4], 80% of the world's cattle population is exposed to tick infestation, among which* Rhipicephalus microplus*, commonly known as “cattle tick,” is widely distributed in most of the countries with tropical and subtropical climate.

The currently available tools for tick control consist of chemical acaricides used with different application methods and various formulations, tick-resistant animals, tick vaccines, and rotations between livestock and crops [4]. Among these, the chemical acaricides form the centre of control and eradication efforts because they offer relatively quick and cost-effective suppression of tick populations. Long-term use, however, has generated acaricide resistance in many tick species [5, 6], thereby reducing the ability to control them. The progressive evolution of resistance of ticks affecting cattle to almost all of the available acaricides has frustrated the efforts of cattle producers to manage TTBDs affecting their animals. It is therefore necessary to look for alternative measures which are adaptable and less expensive, especially for the subsistence farmers with limited capital who constitute the majority of animal rearers in the developing countries, including India. In the recent past various studies have been carried out to develop environmentally safe control measures against ectoparasites and attempts have been made to identify insecticidal/acaricidal properties of different botanical compounds against lice [7], mosquitoes [8], flies [9], ticks [10, 11], and mites [12].

*Ageratum conyzoides* L. (Compositae) is an annual aromatic weed species. It is commonly found in tropical and subtropical regions of Asia, Africa, and South America. In Central Africa it is used to treat pneumonia, but the most common use is to cure wounds and burns. Traditional communities in India use this species as a bacteriocide, antidysenteric, and antilithic [13].* Artemisia absinthium* belongs to Asteraceae family, used in indigenous traditional system of medicine as an antipyretic [14], antimicrobial [15], and antifungal [16]. In Turkish folk medicine, it has been used as an antiseptic, diuretic, and antispasmodic [17]. In India,* A. absinthium* grows throughout the Kashmir valley usually on open mountainous slopes and is locally known as “Tethwen” [18]. To the best of our knowledge, no study was conducted to investigate the acaricidal properties of* A. conyzoides* and* A. absinthium* against* R. microplus*. The present study was therefore designed to evaluate the in vitro acaricidal activity of ethanolic extract obtained from the aerial parts of* A. conyzoides* and* A. absinthium* on* R. microplus* at Jammu, India.

## 2. Materials and Methods

### 2.1. Plant Materials and Extraction

**Figure 1:**
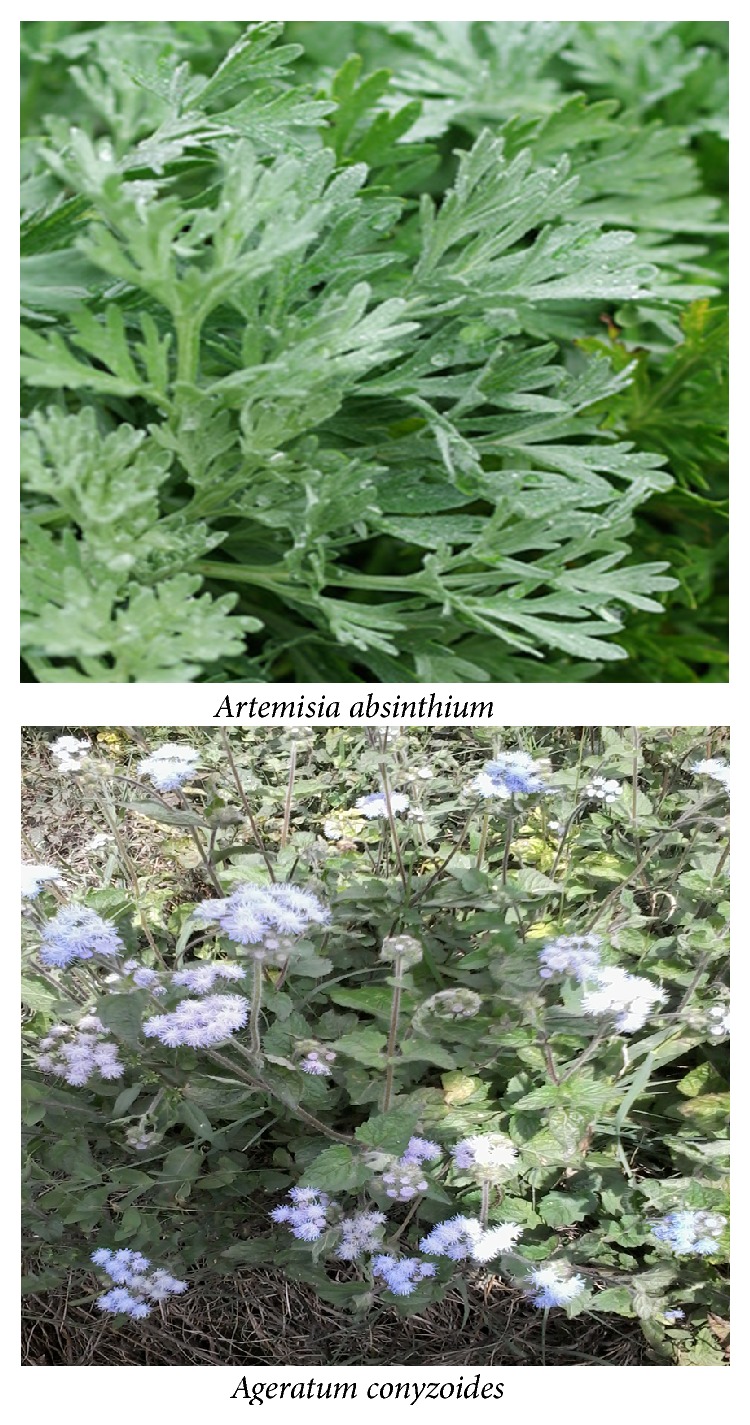
Plants used for extract preparation.

The aerial parts of* A. conyzoides* were collected from R.S. Pura, Jammu, India (Figure 1). The plant was identified by the curator of the Herbarium, Botanical Department, University of Jammu (India). The aerial parts of* A. absinthium* were purchased from Agro Food Processing Emporium, Peerbagh, Srinagar, Jammu and Kashmir, India (Figure 1). The fresh plant materials collected were cleaned of adulterants and air-dried under shade for a period of two weeks and were precrushed in a mortar and later pulverised into fine powder using electric blender. The powder was sieved through a mesh (2 mm size). Sieved powder of plants was used for preparation of ethanolic extracts. For* A. conyzoides*, the extract was prepared by adding 10 g of the plant powder in 200 mL of ethanol in extract container of Soxhlet extractor equipment. The extraction process was done for 24 h at 45–65°C. For* A. absinthium*, the 100 g of powder was soaked in 500 mL of 95% ethanol to exhaustion (~120 h). The extraction was carried out in a percolator by a combination of maceration and percolation at room temperature. The filtrates were collected through a piece of porous cloth. The semisolid viscous masses were dried at 40°C under reduced pressure and a rotation speed of 20 rpm in vacuum rotary evaporator yields the extracts. The extracts were scrapped off, transferred to air tight containers, and stored in a freezer at −20°C till subsequent uses.

### 2.2. Preparation of Stock and Test Concentrations

The ethanolic extracts of* A. conyzoides* and* A. absinthium* were dissolved in dimethyl sulphoxide (DMSO) to prepare stock solutions. Serial dilutions used in the adult immersion test (AIT) were made in distilled water, in order to obtain the concentrations of 1.25%, 2.5%, 5%, 10%, and 20%.

### 2.3. *Rhipicephalus microplus* Ticks for Bioassays

The engorged adult-female ticks dropped from bovines were collected from dairy farms of Jammu (India) and brought to the laboratory in wide mouthed glass jars sealed with muslin cloth. The ticks were thoroughly washed with tap water and dried on filter paper towel. The identification of ticks was made under stereomicroscope according to keys and descriptions described elsewhere [19]. These ticks were used in the AIT within 24 h of collection.

### 2.4. Adult Immersion Test

The AIT was performed as described by Drummond et al. [20] with minor modifications. The ticks were weighed and assigned to groups randomly (10 ticks per group). The different groups of ticks were immersed in 10 mL of the respective concentrations (1.25%, 2.5%, 5%, 10%, and 20%) of* A. conyzoides* and* A. absinthium* by placing them directly into containers and were stirred vigorously with glass rod before and after adding ticks. After 5 min, the acaricides were poured off through a sieve and the ticks were transferred to the tissue paper towel for drying and kept separately in glass tubes and sealed with muslin cloth. For each concentration, three replications were maintained. Simultaneously, the ticks in the control group were treated with 10% DMSO and three replications were maintained. The treated ticks were kept in desiccator which was kept in BOD incubator at a temperature of 27 ± 2°C and relative humidity of 80 ± 5% for oviposition. The mortality was observed on day 14 posttreatment (PT). The ticks which did not oviposit even after 14 days were considered dead. The eggs laid by these ticks were collected, weighed, and observed separately at the same condition of incubation for the next 30 days for visual estimation of hatching rate.

The percentage inhibition of oviposition (IO) was calculated as follows:

reproductive index (RI) = average weight of eggs laid (mg)/average weight of live ticks (mg):

(1) $$\begin{array}{c} \text{IO}\left(\%\right)=\frac{\text{RI}\;\;\text{of}\;\;\text{control}\;\;\text{ticks}-\text{RI}\;\;\text{of}\;\;\text{treated}\;\;\text{ticks}}{\text{RI}\;\;\text{of}\;\;\text{control}\;\;\text{ticks}}\times 100. \end{array}$$

### 2.5. Statistical Analyses

The lethal concentrations (LC_50_ and LC_95_) and their respective 95% confidence intervals (CI) were determined by applying regression equation analysis to the probit transformed data of mortality. The dose-response data were analysed by probit method [21] using Graph Pad Prism 4 software. A value of *P* < 0.05 was considered significant.

## 3. Results

**Figure 2:**
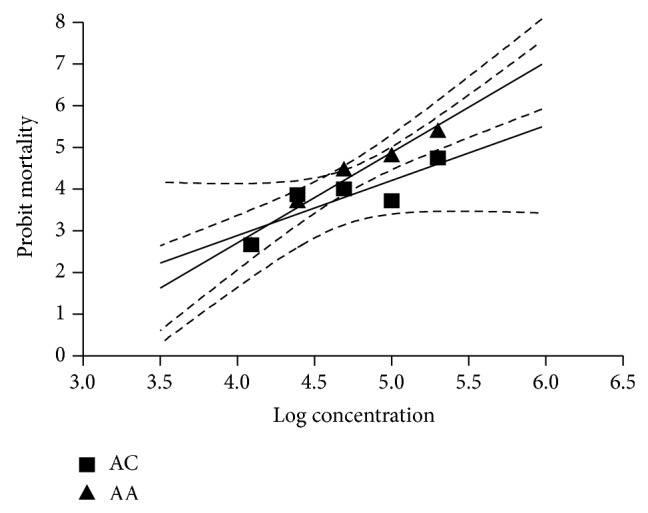
Regression line of probit mortality of* R. microplus* against the log concentrations of the ethanolic extracts of* A. conyzoides* (AC) and* A. absinthium* (AA) using AIT.

The results of adult immersion test for* A. conyzoides* and* A. absinthium* are presented in Tables 1 and 2, respectively. The maximum mortality of 40.0% for* A. conyzoides* was recorded at 20% concentration. However, it was statistically not significant (*P* > 0.05) in comparison to the control group, whereas the maximum mortality of 66.7% was recorded for* A. absinthium* at 20% concentration and it was statistically significant (*P* < 0.05) in comparison to the control group. The regression graph of mean mortality of ticks plotted against values of concentration is shown in Figure 2. From the regression equation the LC_50_ (CI) and LC_95_ (CI) values were calculated as 34.36% (9.82–120.3) and 537.4% (392.00–736.7) for* A. conyzoides* and 11.22% (10.39–12.1) and 61.77% (19.59–194.5) for* A. absinthium*, respectively (Table 3). The mortality slope values and *R* ^2^ (goodness of fit) values for* A. conyzoides* and* A. absinthium* are given in Table 3.

As the cut-off date for observation of adult mortality for both control and treated ticks was day 14, the egg mass laid up to day 14 was considered under this study. It is important to note that the live ticks which were treated with different concentrations of the ethanolic extracts laid egg masses which were significantly (*P* < 0.05) lower in weight than the egg masses of the control ticks, despite there being low percent adult mortality in the treated ticks, mainly for* A. conyzoides*. Further, a significant decrease (*P* < 0.05) in the RI was recorded in all the concentrations of* A. conyzoides* and* A. absinthium *extracts in comparison to the control ticks. Inhibition of oviposition observed in AIT varied from 22.6 to 60.6% for* A. conyzoides* and 6.7 to 65.9% for* A. absinthium*. The effect on the hatchability of eggs of treated groups when compared to the control was highly variable and the complete inhibition (100%) was recorded at 5%, 10%, and 20% concentrations of the extract of* A. absinthium. *The mortality slope values and *R* ^2^ values of egg mass and RI and IO for* A. conyzoides* and* A. absinthium* are given in Table 3.

## 4. Discussion

In the current study, we evaluated the in vitro acaricidal activity of ethanolic extract of* A. conyzoides* and* A. absinthium *against engorged adult females of* R. microplus *by measuring mortality, IO, and hatching rate. In the present study, the extract of* A. conyzoides* at 5% concentration caused 16.7% mortality of ticks, but at 10% the extract dilution was not good, thereby explaining its lesser efficacy (10.0%) because of less bioavailability. Only few reports are available regarding acaricidal activity of* Ageratum *species against ticks [22, 23], and to the best of our knowledge no published data are available on the effect of extract of* A. conyzoides* against* R. microplus*. It has been reported that the terpenic compounds, mainly Precocene I and Precocene II, are the main components of* Ageratum *species [22, 24]. The Precocenes are highly specific chemical substances, which attack certain areas of the insect endocrine system not only causing toxic effects but also disturbing the development process and reproduction. Further, these compounds have been shown to induce morphogenetic abnormalities in the formation of insects. They accelerate larval metamorphosis, resulting in intermediary stages between larvae-pupae, discolored and longer pupae, and incompletely developed adults or sterile adults [25, 26]. Booth et al. [27] demonstrated that the treatment of engorged females of* Boophilus microplus* with Precocene resulted in desiccated nonviable eggs due to absence of a waterproofing wax layer which is mandatory for proper hatching of eggs and maintenance of fecundity of ticks. In an experiment with* Ornithodoros moubata* females treated with Precocenes I and II, it was observed that the oviposition was reduced in ticks that survived repeated treatments with Precocene II [28]. Pamo et al. [22] evaluated the acaricidal effect of the essential oil of the flowers of* Ageratum houstonianum* on* Rhipicephalus lunulatus*. They observed concentration dependent increase in mortality rate which also increased in the course of time (day), reaching a maximum of 100% with the dose of 0.125 *μ*L/cm^2^ on fifth day of treatment. Again, Pamo et al. [23] assessed the in vitro and in vivo acaricidal effect of foam soap containing the essential oil of* A. houstonianum *leaves on* R. lunulatus. *They recorded 100% mortality rate in the in vitro experiment with the dose of 0.03 mL/g on day 3 after treatment, whereas in the in vivo experiment, 95.1% mortality was recorded on day 8 after treatment. The LD_50_ of the foam soap containing essential oil was 0.0259 and 0.0173 mL/g on day 2 after treatment, in the laboratory and on the farm, respectively.

*Artemisia absinthium* is a rich source of terpenes, antioxidant phenolics, flavonoids, and other biologically active compounds [29]. In modern medicine, these compounds have been investigated for their anthelmintic and antioxidant activities in parasitised animals by neutralizing the free radicals and toxins formed in their blood, boost their immune system, and help in fighting against parasites [30]. In our previous study [10], the chloroform extract obtained from the aerial parts of* A. absinthium* showed 93.3% mortality rate against adults of* R. sanguineus* at 20% concentration with the LC_50_ and LC_95_ values of 8.79% and 34.59%, respectively. Besides a significant reduction (*P* < 0.05) in the egg mass (85.1%) at 20% concentration and complete blockage of egg hatchability, 100% larval mortality at 5% concentration of the extract has also been observed. Chagas et al. [31] reported greater efficacy of the extracts of* A. annua* against* R. *(*B.*)* microplus* with EC_50_ of 130.6 mg/mL and EC_90_ of 302.9 mg/mL. However, the extracts were not effective on the larvae of* R. *(*B.*)* microplus* at the concentrations tested (3.1 to 50 mg/mL). Earlier, the mode of action of artemisinin, the active component of* Artemisia* spp., has been investigated in trematodes, gastrointestinal nematodes, monogeneans, and* R*. (*B*.)* microplus* by various workers [31–33]. The artemisinin is thought to exert its effect by reacting with the heme groups of the haemoglobin molecules digested by parasites, altering the cell structure and its functions through the free radicles derived from artemisinin, thus affecting the growth and reproduction [34].

In the Indian subcontinent, the environmental conditions are suitable for faster propagation and survival of ixodid tick species.* Rhipicephalus microplus* is a common tick species infesting dairy animals throughout the country [35]. Recently, the presence of widespread resistance to synthetic pyrethroids in* R. microplus* has been reported from the different parts of the country [5, 6, 36, 37]. The natural compounds derived from plants are more stable as these are mostly plant secondary metabolites synthesized over a long period of time. Moreover, the natural compounds also provide greater structural diversity than synthetic ones and therefore are a source of low molecular weight structures active against a wide range of target agents and this diversity can preclude the occurrence of resistance. Based on the results of the current study, it can be concluded that* A. conyzoides* and* A. absinthium* could form alternatives to commercially available synthetic acaricides; however, further investigations against different tick species and its actual in vivo application are required to determine the true potential of* A. conyzoides* and* A. absinthium *as effective herbal formulations for the control of tick infestation on animals.

## Figures and Tables

**Table 1:** Tick weight, percent mortality, egg weight, reproductive index (RI), and inhibition of oviposition (IO) of *R. microplus* adults exposed to different concentrations of *A. conyzoides*.

Conc.	Live tick wt. (mg)	Mortality (%)	Egg wt. (mg)	RI	IO (%)	Hatching rate (%)
(%)	(Mean ± SE)	(Mean ± SE)	(Mean ± SE)	(Mean ± SE)	(Mean ± SE)	
Control	113.2 ± 5.75	0.0 ± 0.0	64.9 ± 3.7	0.566 ± 0.0	0.0 ± 0.0	100
1.25	110.2 ± 4.45	0.0 ± 0.0	48.2 ± 2.84	0.428 ± 0.02	22.6 ± 7.54	75
2.5	105.7 ± 3.91	15.0 ± 5.0	45.8 ± 4.71	0.437 ± 0.04	24.4 ± 3.58	25
5	98.5 ± 4.35	15.0 ± 9.57	33.5 ± 3.92	0.363 ± 0.04	36.3 ± 7.37	20
10	101.5 ± 4.23	10.0 ± 5.77	36.4 ± 3.96	0.370 ± 0.04	34.6 ± 6.91	20
20	103.6 ± 3.36	40.0 ± 11.54	23.4 ± 4.16	0.223 ± 0.05	60.6 ± 8.39	5
*P* value		0.0844	0.0210	0.0489	0.0388	

**Table 2:** Tick weight, percent mortality, egg weight, reproductive index (RI), and inhibition of oviposition (IO) of *R. microplus* adults exposed to different concentrations of the ethanolic extract of *A. absinthium*.

Conc.	Live tick wt. (mg)	Mortality (%)	Egg wt. (mg)	RI	IO (%)	Hatching rate (%)
(%)	(Mean ± SE)	(Mean ± SE)	(Mean ± SE)	(Mean ± SE)	(Mean ± SE)	
Control	114.9 ± 5.4	0.0 ± 0.0	54.1 ± 3.8	0.470 ± 0.0	0.0 ± 0.0	100
1.25	101.0 ± 5.6	0.0 ± 0.0	44.3 ± 2.7	0.439 ± 0.1	6.7 ± 1.7	45
2.5	96.0 ± 7.3	10.0 ± 5.81	33.8 ± 4.9	0.366 ± 0.04	13.8 ± 4.4	5
5	90.0 ± 8.9	30.0 ± 5.82	22.4 ± 6.2	0.239 ± 0.06	27.3 ± 6.37	0
10	91.0 ± 6.2	43.3 ± 8.8	15.8 ± 4.3	0.180 ± 0.05	36.2 ± 6.1	0
20	113.0 ± 13.5	66.7 ± 6.7	7.5 ± 9.01	0.064 ± 0.03	65.9 ± 3.2	0
*P* value		0.0033	0.0005	0.0005	0.0085	

**Table 3:** Dose-dependent response of *A. conyzoides* (AC) and *A. absinthium* (AA) against *R. microplus* using AIT.

Plant	Variables	Slope ± SE	*R* ^2^	LC_50_% (95% CI)	LC_95_% (95% CI)
AC	Mortality	1.290 ± 0.5071	0.6833	34.36 (9.82–120.3)	537.4 (392.00–736.7)
	Egg mass	−19.44 ± 4.358	0.8690		
	RI	−0.1573 ± 0.04901	0.7746		
	% IO	28.43 ± 8.062	0.8056		

AA	Mortality	2.170 ± 0.2518	0.9611	11.22 (10.39–12.1)	61.77 (19.59–194.5)
	Egg mass	−30.22 ± 1.810	0.9893		
	RI	−0.3088 ± 0.01865	0.9892		
	% IO	46.50 ± 7.525	0.9272		

*R* ^2^: goodness of fit; RI: reproductive index; IO: inhibition of oviposition.
